# Contribution of captured retroviral envelope genes, the "synctins" to the formation of the mouse placenta

**DOI:** 10.1186/1742-4690-8-S2-O24

**Published:** 2011-10-03

**Authors:** Anne Dupressoir, Cécile Vernochet, Francis Harper, Gérard Pierron, Justine Guégan, Philippe Dessen, Thierry Heidmann

**Affiliations:** 1 CNRS UMR 8122, Institut Gustave Roussy, Villejuif 94805, France and Université París-Sud, 91405 Orsay, France; 2 Unité de Génomigue Fonctionnelle, Institut Gustave Roussy, Villejuif 94805, France

## Background

In most mammalian species, a key process of placentation is the fusion of trophoblast cells into a multinucleated syncytiotrophoblast layer at the fetomaternal interface. Envelope proteins of retroviral origin, the syncytins, specifically expressed in the placenta and with *in vitro* cell-cell fusogenic activity, have been suspected to be involved in this process [1-5]. In mice, inactivation of *syncytin-A* resulted in impaired formation of the syncytiotrophoblast layer I (facing the maternal blood lacuna) and in embryonic death [6], demonstrating that it is required for placenta development. Here, the effect of the inactivation of the second murine *syncytin* gene, *syncytin-B*, is described.

## Materials and methods

We generated *syncytin-B* mutant mice, and embryos homozygous mutant for *syncytin-B* or for both *syncytin-A* and -*B* were obtained at different gestational stages, through time mating intercrosses. The placentae of *syncytin-B* null embryos were examined using histological analyses and refined electron microscopy analyses of the fetomaternal interface. Genes whose expression is modified in null placenta were identified using microarrays analyses and their expression was localized by immunohistochemistry.

## Results

*Syncytin-B* null placenta disclose defects in formation of the syncytiotrophoblast layer II (facing the fetal blood vessels), with enlarged maternal lacuna disrupting the placenta architecture. At variance with the *syncytin-A* phenotype, *syncytin-B* null embryos are viable, although they still display late-onset embryonic growth retardation and a significant perinatal death rate. Interestingly, double SynA/SynB KO embryos die earlier than do embryos deficient for *syncytin-A* alone, suggesting cooperative interactions. Finally, an induction of *connexin-30* expression was observed in the *syncytin-B* null placenta, with the protein being detected at the fetomaternal interface, suggesting a compensatory process whereby impaired syncytialization is counteracted by a gap junction mediated, cell-cell communication.

## Conclusions

These findings demonstrate that *syncytin-B* is essential for syncytiotrophoblast formation and placenta integrity. Altogether, our data demonstrate that the two murine *syncytins* contribute independently to the formation of the two syncytial layers. Placenta formation is therefore a complex process that has taken advantage of independent and stochastic "captures" of retroviral sequences in the course of evolution to generate "appropriate" structures and functions. Some of them are absolutely required for pregnancy, whereas the other could still be amenable to compensatory processes.
